# Femorotibial Bypass Sutured on Bovine Pericardium Patch of the Femoral Artery for Bypass Inflow: A Case Report

**DOI:** 10.7759/cureus.49381

**Published:** 2023-11-25

**Authors:** Sakamoto Daisuke, Sakamoto Takuya, Nagayoshi Yasuhiro, Takano Tamaki

**Affiliations:** 1 Cardiovascular Surgery, Kanazawa Medical University Hospital, Ishikawa, JPN; 2 Medical Research Institute, Kanazawa Medical University, Ishikawa, JPN; 3 Cardiovascular Surgery, Kanazawa Medical University, Ishikawa, JPN

**Keywords:** atherosclerosis, bovine pericardium, thromboendarterectomy, femorotibial bypass, critical limb ischemia

## Abstract

A 72-year-old man presented with intermittent claudication and a foot ulcer. Computed tomography revealed severe calcification and occlusion of the left femoral artery and calcification and stenosis from the superficial femoral artery to the popliteal artery. Thromboendarterectomy (TEA) and anterior reconstruction of the femoral artery with a bovine pericardium patch were performed. We sutured the great saphenous vein on the bovine pericardium patch for bypass inflow after creating an anastomosis hole with a puncher and performed an in situ femorotibial bypass. This technique helped us achieve a smooth and clean anastomosis. In situ vein graft anastomosis might be difficult on severely atherosclerotic femoral artery after TEA and difficult anastomosis increases the risk of bypass occlusion. Anastomosis on the bovine pericardium patch for bypass inflow might ensure smooth and clean anastomosis in patients with severe atherosclerosis of the femoral artery.

## Introduction

Femorotibial bypass is an effective treatment for critical limb ischemia (CLI) and accounts for 54% of surgical reconstructive procedures as per the 2019 Japan Critical Limb Ischemia Database annual report [1]. However, atherosclerotic changes (e.g., calcification and intimal hyperplasia) of the femoral artery in the femorotibial bypass may complicate anastomosis for bypass inflow and increase the risk of graft occlusion [2]. Bovine pericardium patch (XenoSure; LeMaitre Vascular, Inc., Burlington, MA) became available in Japan for thromboendarterectomy (TEA) of the femoral artery in April 2020 because it demonstrated favorable outcomes in carotid endarterectomy [3]. Herein, we used a bovine pericardium patch for TEA of the femoral artery and performed a femorotibial bypass on the bovine pericardium at the inflow. This technique would allow easier anastomosis at the bypass inflow in patients with severe atherosclerotic changes of the femoral artery.

## Case presentation

**Figure 1:**
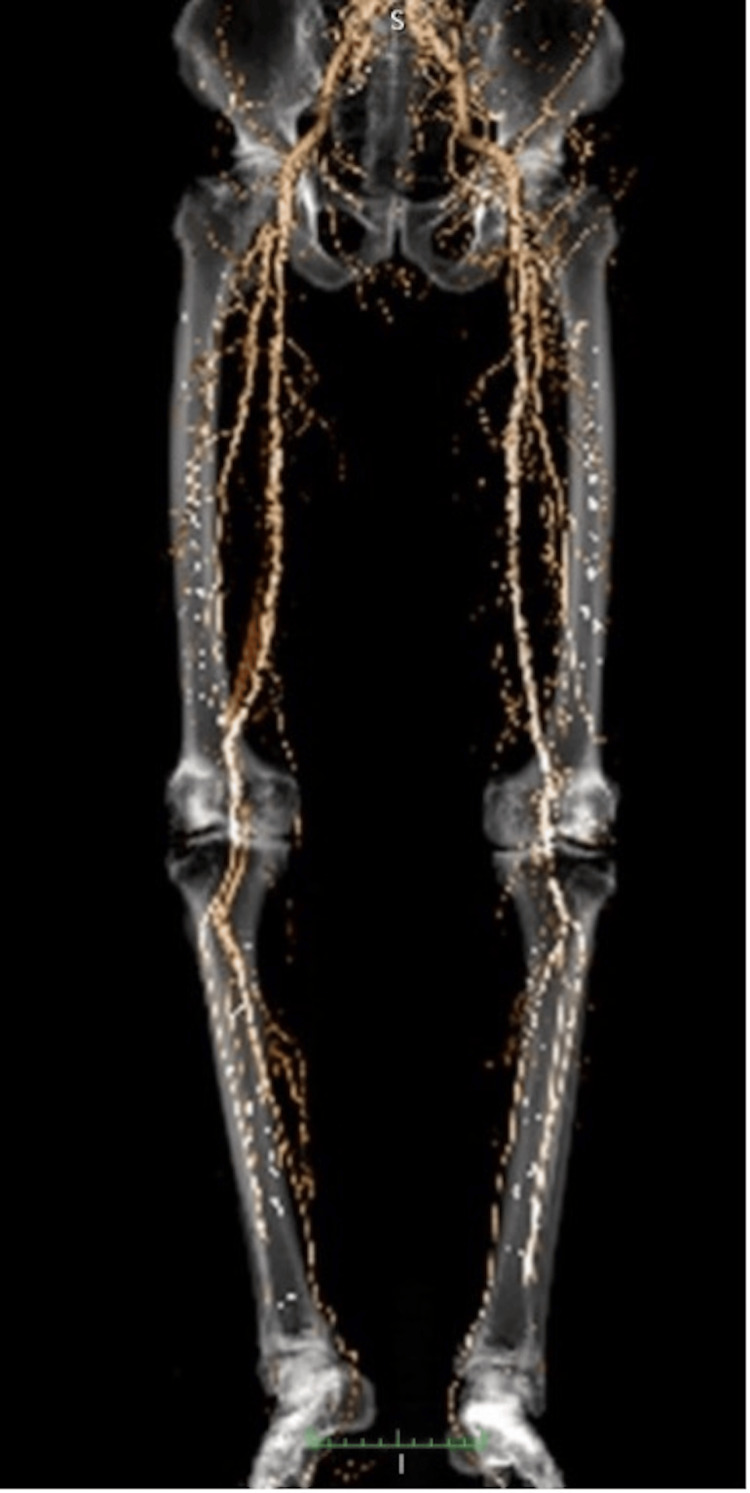
Preoperative computed tomography angiogram revealed severe calcification and occlusion from the common femoral artery to the femorotibial popliteal artery.

A 72-year-old man who presented with intermittent claudication of the left limb was referred to our hospital for surgery. He was diagnosed with nephrosclerosis resulting in renal failure and started hemodialysis at the age of 62 years. He had a smoking history until the age of 70 and received percutaneous coronary intervention for angina pectoris to the right coronary artery. A stent was also percutaneously placed in the right common iliac artery for CLI. He had ulceration on the hallux. No pulse could be palpated from the left femoral to the posterior tibial arteries. The ankle-brachial index (ABI) was significantly decreased on the left side (p = 0.48). Contrast-enhanced computed tomography (CT) showed severe calcification and occlusion of the left femoral artery and calcification and stenosis from the superficial femoral artery to the femorotibial popliteal artery (Figure 1).

**Figure 2:**
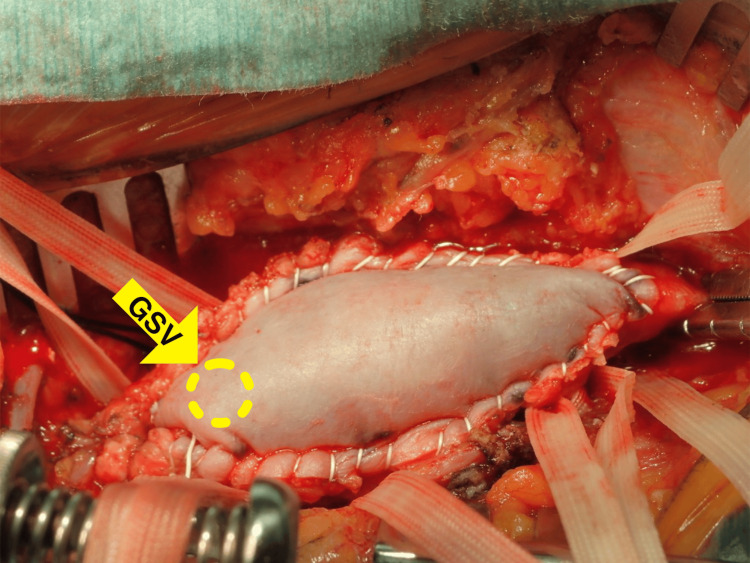
An endarterectomy of 7 cm in length was carefully performed and a bovine pericardium patch was used. The yellow arrow shows a 4.5-mm hole punched in the patch and the great saphenous vein (GSV) graft was sutured.

The patient was clinically diagnosed with stage 3 severe left limb ischemia (Fontaine IV, Rutherford 5), hence in situ femorotibial bypass was performed under general anesthesia. First, all great saphenous vein (GSV) branches were ligated through small incisions under ultrasound guidance. The femorotibial end of GSV, posterior tibial artery, and the proximal end of the femoral artery and GSV were exposed. The femoral artery was severely calcified and occluded, hence TEA was performed ordinarily. The diameter of the femoral artery was 10 mm, and covering the anterior femoral artery’s wall defect with GSV was considered unfavorable. Therefore, the incision was extended to the deep femoral artery bifurcation and XenoSure was continuously sutured using CV-5 (GORE-TEX; W.L. Gore & Associates, Inc., Newark, DE). Then, a small hole was created on XenoSure using a 4.5-mm puncher, and the proximal end of GSV was sutured continuously using 7-0 polypropylene (PROLENE; Ethicon, Inc., Raritan, NJ) (Figure 2).

**Figure 3:**
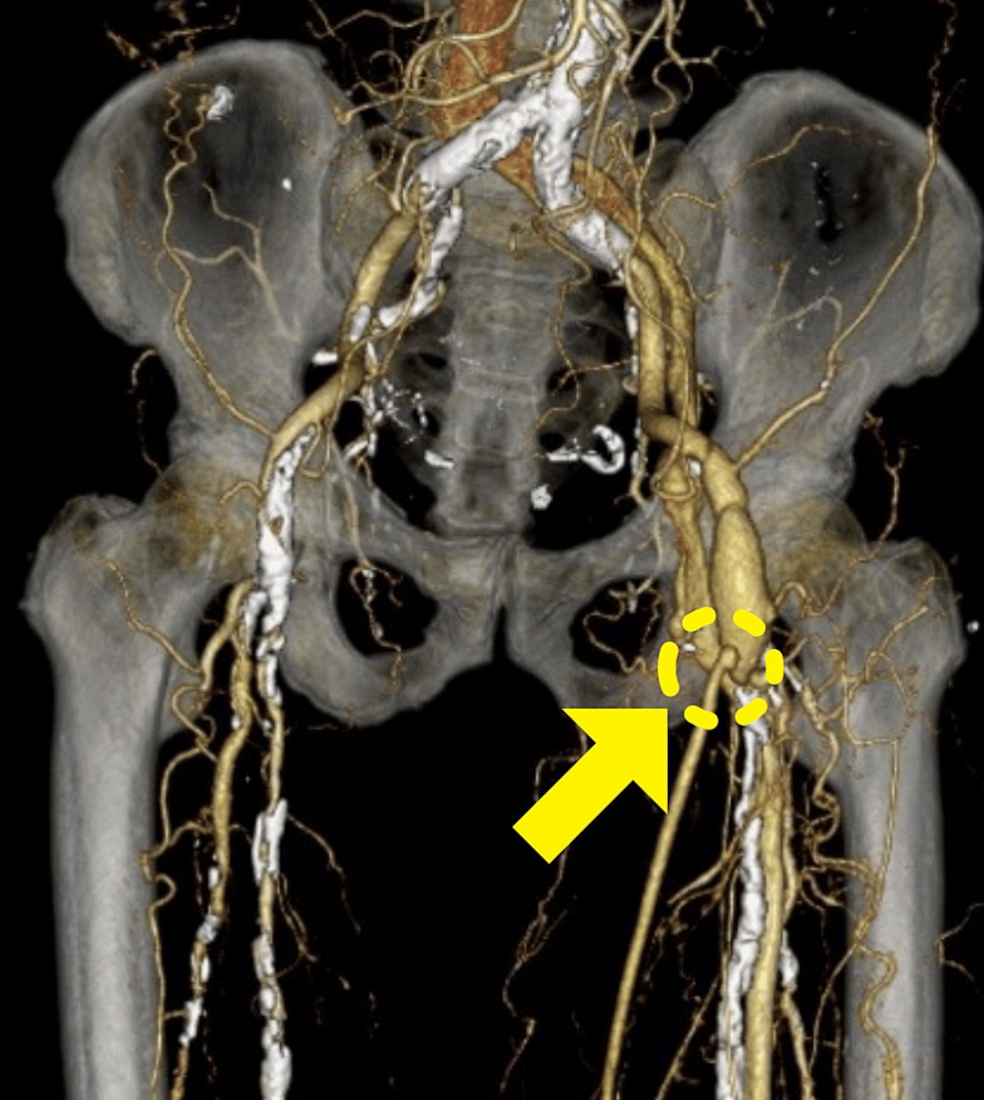
Postoperative computed tomography angiogram revealed the bovine pericardium patch (XenoSure®) plasty from the common femoral artery to the deep femoral artery and great saphenous vein graft on the bovine pericardium patch (yellow arrow).

**Figure 4:**
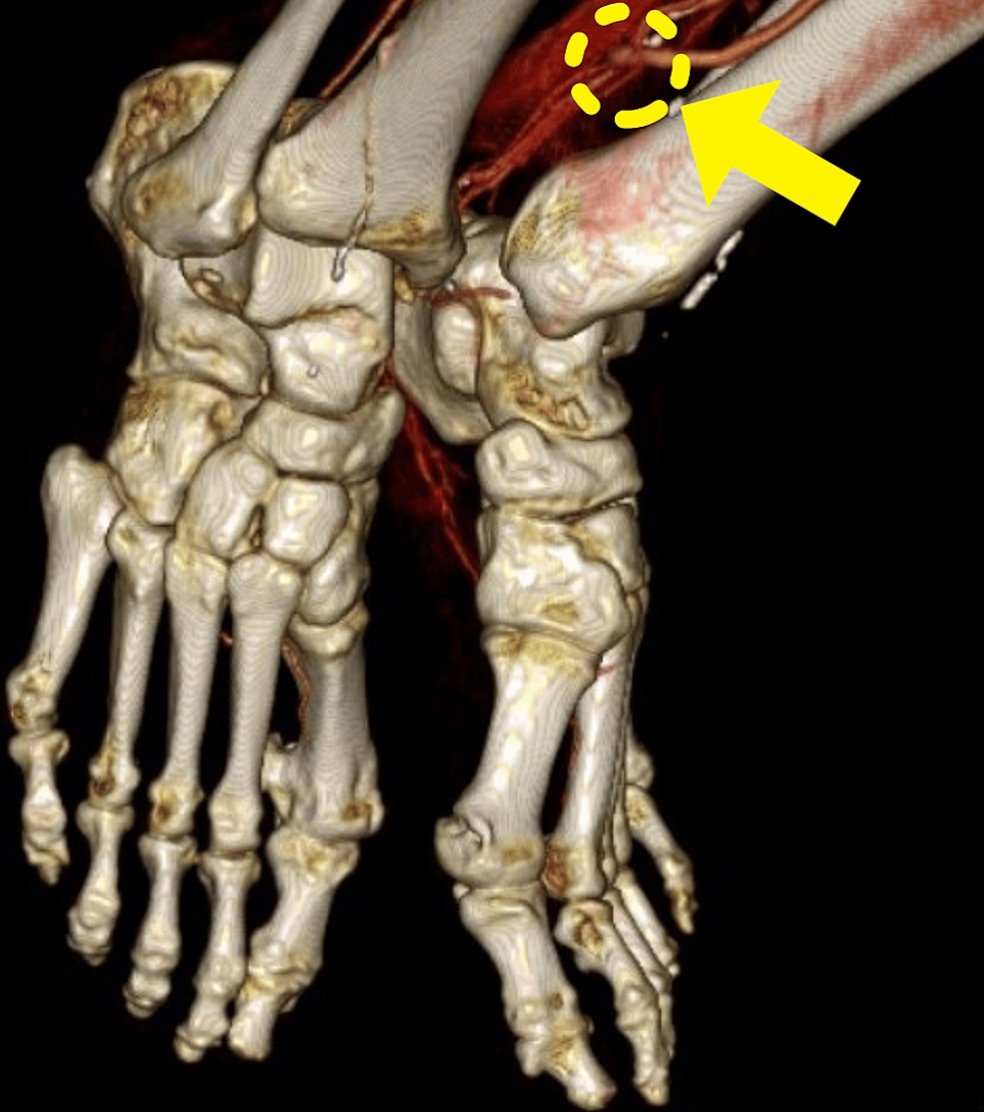
The yellow arrow shows femorotibial anastomosis and runs off the posterior tibial artery.

To confirm antegrade blood flow, venous valves were destroyed from the peripheral side with a venous valve cutter (Valvulotome; LeMaitre Vascular, Inc., Burlington, MA). Finally, GSV was sutured to the posterior tibial artery using 7-0 polypropylene, and blood flow was confirmed by ultrasonography. Intermittent claudication disappeared and ABI was 0.97 post surgery. Graft patency was confirmed by contrast-enhanced CT (Figures 3, 4).

The patient was discharged on postoperative day eight, and no sign of restenosis or obstruction was observed during the one-year follow-up.

## Discussion

Femorotibial bypass provides good blood flow to the lower limb and restenosis and reoperation are less frequent compared with endovascular treatment in the long term [4], although it is now widely applied for CLI because of its less invasiveness and shorter hospital stay. The reported five-year graft patency rate of the femoral-popliteal, tibial, and peroneal bypass with in situ GSV was 77%, and it is the procedure of choice for long infrapopliteal bypass [5]. In this case, femorotibial bypass with in situ vein graft was performed for the popliteal artery occlusion. The postoperative course was uneventful, and graft patency was favorable; although the observation period was only one-year post surgery.

Herein, a bovine pericardium patch was used to cover the anterior wall of the femoral artery after the removal of calcified and sclerotic intima. Saphenous vein patch reconstruction in TEA has been widely used since the initial case reported in 1964 [6]. Compared with vein patch closure, direct closure results in more perioperative stenosis and dilatation [7]. The bovine pericardium patch showed better patency rates than the saphenous vein patch in carotid endarterectomy and more recently in femoral endarterectomy [8,9]. It also resulted in lesser bleeding from the anastomotic site and fewer complications than conventional artificial vessel graft or venous patch [10]. When bypass is performed with in situ GSV, another GSV harvest is required for the TEA patch. A bovine pericardium patch is considered useful for calcification and severe atherosclerosis of the femoral artery, as observed in this case. However, the foreign nature of bovine pericardium may result in infectious complications, although the incidence rate did not show a significant difference as compared to venous patches [9]. Careful observation is required after TEA with a bovine pericardium patch.

We sutured GSV on the bovine pericardium patch for bypass inflow after creating an anastomosis hole with a puncher and performed an in situ femorotibial bypass. This technique helped us achieve a smooth and clean anastomosis. In situ vein graft anastomosis might be difficult on severely atherosclerotic femoral artery after TEA and difficult anastomosis increases the risk of bypass occlusion. Postoperative CT showed good patency and the patient had no signs of graft stenosis and occlusion one year post surgery. Our method of anastomosis on the bovine pericardium patch might be useful for proximal anastomosis of the in situ femorotibial bypass.

## Conclusions

Femorotibial bypass is an effective treatment for CLI. However, atherosclerotic changes of the femoral artery in the femorotibial bypass may complicate anastomosis for bypass inflow and increase the risk of graft occlusion. We used a bovine pericardium patch for TEA of the femoral artery and performed a femoropopliteal bypass on the bovine pericardium at the inflow. Anastomosis on the bovine pericardium patch for the bypass inflow might ensure smooth anastomosis in patients with severe femoral artery atherosclerosis.
